# Pathologic fracture of the distal radius in a 25-year-old patient with a large unicameral bone cyst

**DOI:** 10.1186/1471-2474-15-202

**Published:** 2014-06-13

**Authors:** Felix Massen, Sebastian Baumbach, Elias Volkmer, Wolf Mutschler, Stefan Grote

**Affiliations:** 1 Department of Trauma Surgery, Ludwig-Maximilians-University, Nussbaumstrasse 20, 80336 Munich, Germany

**Keywords:** Distal radius fracture, Pathological fracture, Unicameral bone cyst, Fallen fragment sign, Operative treatment, Arthroscopic reduction, Bone graft, RIA

## Abstract

**Background:**

Distal radius fractures (DRF) are often referred to as osteoporosis indicator fractures as their incidence increases from age 45. In the group of young adults, distal radius fractures normally result from high-energy trauma. Wrist fractures in young patients without adequate trauma thus raise suspicion of a pathologic fracture. In this report we present the case of a fractured unicameral bone cyst (UBC) at the distal radius in a young adult.

To the author’s best knowledge, this is the first detailed report in an UBC at the distal radius causing a pathologic DRF in an adult patient.

**Case presentation:**

A 25-year-old otherwise healthy male presented to our Emergency Department after a simple fall on his right outstretched hand. Extended diagnostics revealed a pathologic, dorsally displaced, intra-articular distal radius fracture secondary to a unicameral bone cyst occupying almost the whole metaphysis of the distal radius. To stabilize the fracture, a combined dorsal and volar approach was used for open reduction and internal fixation. A tissue specimen for histopathological examination was gathered and the lesion was filled with an autologous bone graft harvested from the ipsilateral femur using a reamer-irrigator-aspirator (RIA) system. Following one revision surgery due to an intra-articular step-off, the patient recovered without further complications.

**Conclusions:**

Pathologic fractures in young patients caused by unicameral bone cysts require extended diagnostics and adequate treatment. A single step surgical treatment is reasonable if fracture and bone cyst are treated appropriately. Arthroscopically assisted fracture repair may be considered in intra-articular fractures or whenever co-pathologies of the carpus are suspected.

## Background

Distal radius fractures (DRF) are among - if not the - most common fractures. In adults, they are often referred to as osteoporosis indicator fractures because their incidence increases from age 45. There is a second incidence peak between the age of 8 to 14 with a male predominance. The etiology differs significantly between the two age groups. While osteoporosis-related DRF often result from inadequate trauma, fractures in adolescents or young adults usually arise from high energy trauma such as car or sports accidents [1,2]. Consequently, a DRF after a low-energy impact in young individuals should always raise suspicion of a pathologic fracture.

## Case presentation

### Initial presentation and diagnostics

A 25-year-old otherwise healthy male presented to our Emergency Department after a simple fall on his right outstretched hand. A thorough history did not reveal any predisposing risk factors, previous fractures or tumors. Upon clinical examination, there was a severe swelling and tenderness of the right wrist with a reduced range of motion.

**Figure 1:**
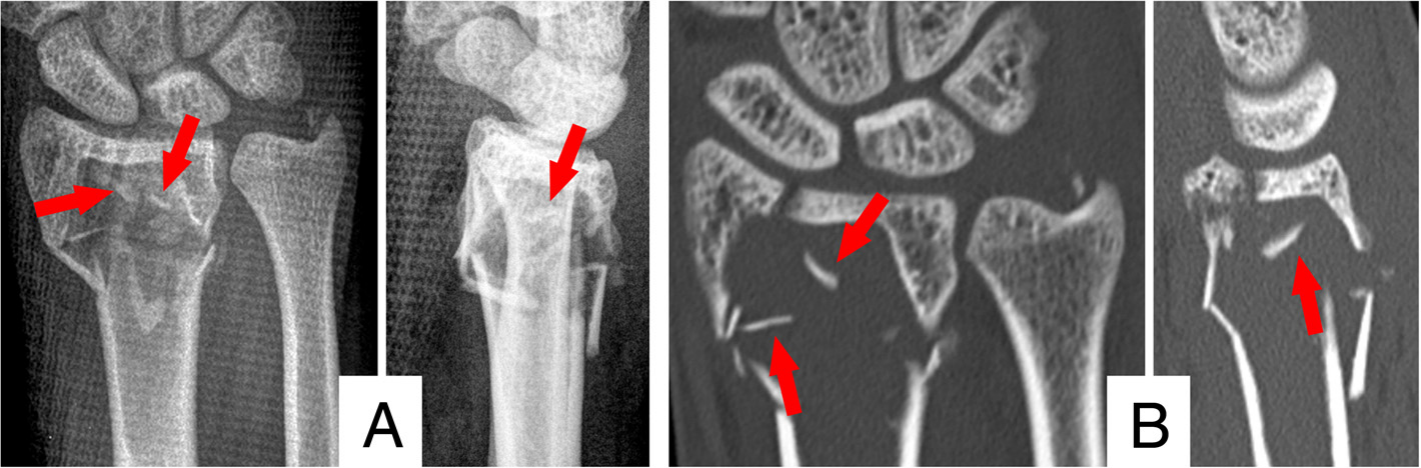
**Summary of the radiographic findings. A)** Plain radiographs of the wrist in two planes; **B)** Computed tomography of the distal radius; red arrows mark the ’fallen fragment sign’.

Plain radiographs showed a dorsally displaced, intra-articular distal radius fracture. The distal metaphysis was almost entirely substituted by a cyst-like lesion containing free bony fragments, suggesting the ‘fallen fragment sign’. The subsequent computed tomography (CT; Discovery CT 750HD; GE Healthcare, Waukesha, WI, USA) confirmed a pathologic DRF AO type 23-C2 as a result of a bone cyst. The lesion was surrounded by a sclerotic rim measured 3.8 × 2.3 × 1.7 cm (length × width × height) and displayed a cyst index of 3.77 [3]. The intra-cavity density ranged between 40–55 Hounsfield Units (resembling blood). The radiographic findings are summarized in Figure 1. Taken together, the lesion was highly suggestive of a unicameral bone cyst (UBC). Differential diagnoses included an aneurysmatic bone cyst (ABC), a giant cell tumor (GCT) or a non-ossifying fibroma.

### Treatment

**Figure 2:**
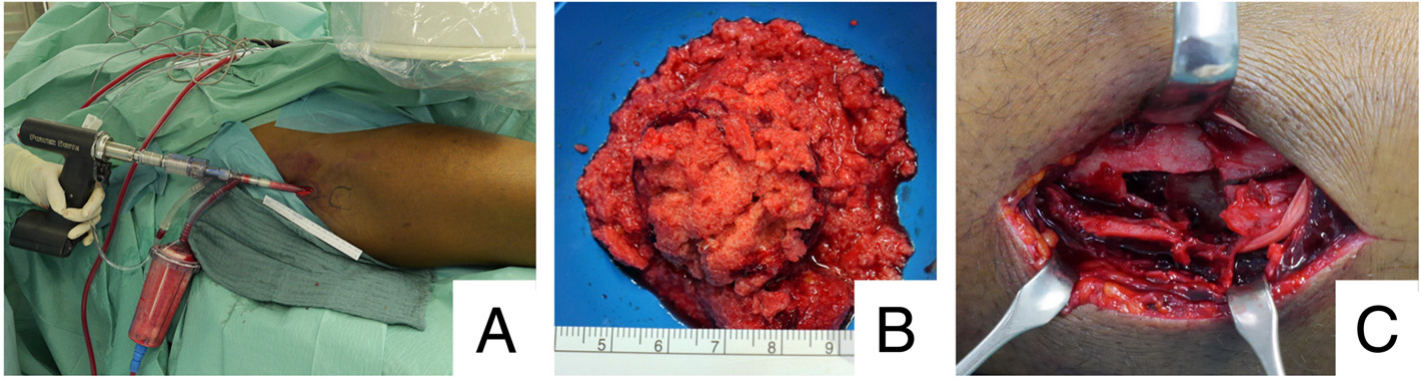
**Intraoperative images of intramedullary bone graft harvesting, cyst size and filling. A)** Intraoperative Setup of the RIA; **B)** bone harvest gathered by RIA; **C)** illustration of the dorsal approach showing the comminuted zone as well as the bone cyst.

**Figure 3:**
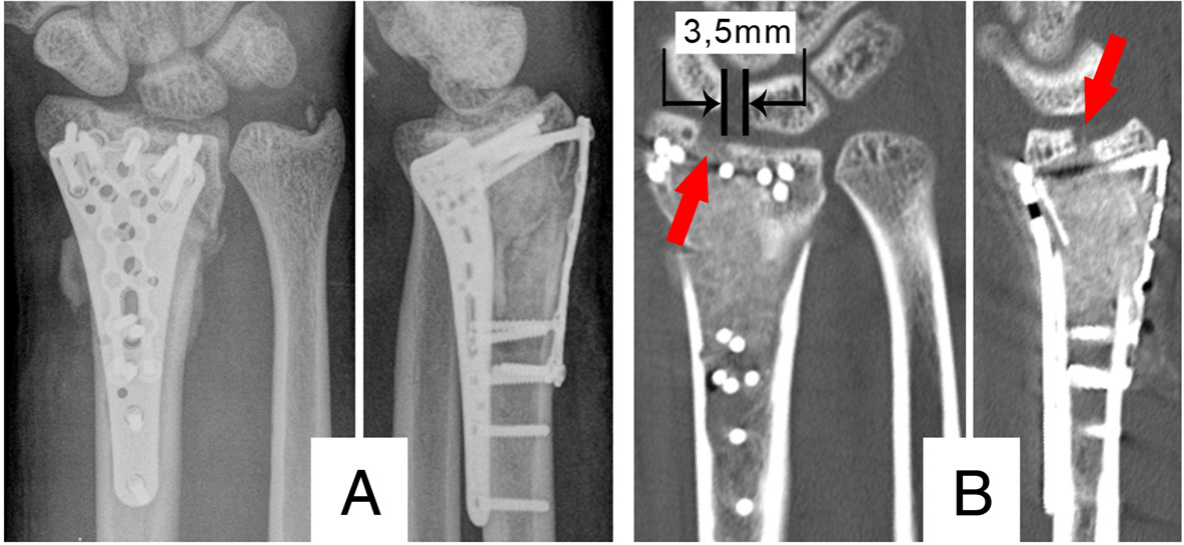
**Postoperative computed tomography. A)** Postoperative radiographs; **B)** postoperative computed tomography, the red arrows show a 2-3 mm intra articular gap and the scapholunate ligament dissociation of 3,5 mm is marked.

**Figure 4:**
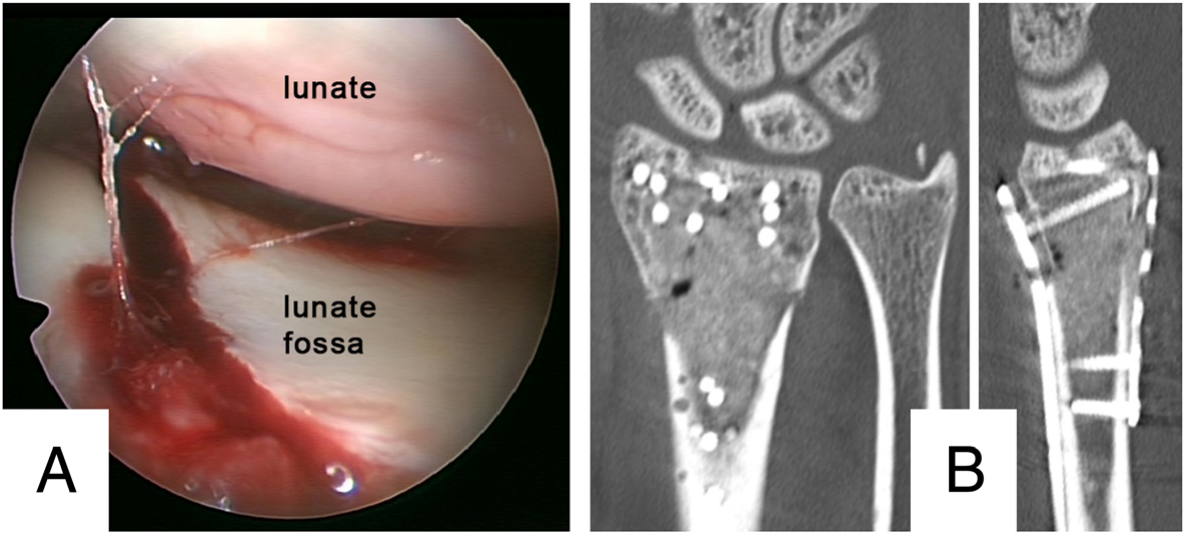
**Perioperative arthroscopic images and postoperative CT-scan. A)** Perioperative image of the arthroscopy showing the articular surface after mobilization and repositioning of the ulnar and radial fragment; **B)** postoperative CT scan with good joint alignment.

Because the tumor was suspected to be benign, we carried out surgical treatment without prior biopsy. Firstly, autologous intramedullary bone graft harvesting was performed at the ipsilateral femur with a reamer-irrigator-aspirator system (RIA, Synthes, Umkirch, Germany). The intraoperative setup and gathered bone graft are illustrated in Figure 2A and B. Secondly, the unicameral bone cyst was thoroughly debrided via a combined volar and dorsal approach to the distal radius. After collection of histological specimen, the anatomy of the radius was restored by open reduction and internal fixation using an angular stable volar plate (Distal Radius Plate, Aptus, Medartis, Basel, Switzerland). We then filled the debrided bone cyst (Figure 2C) with the harvested bone graft through the dorsal approach. To restrain the dorsal fragments and bone graft, a small double-rowed 6-hole plate (Aptus Hand Plate, Medartis, Basel, Switzerland) was used.Although the postoperative radiographs and CT scan revealed a complete filling of the bone cyst, it showed an intra-articular step of 2-3 mm as well as a distended scapholunate (SL) interval indicative of an SL-ligament tear (Figure 3).Therefore, arthroscopically assisted revision surgery was scheduled four days later. Despite the apparent SL distension, the SL-ligament was intact. Under arthroscopic guidance, the screws of the radial and intermediate column of the volar plate were loosened, the gap smoothened and the screws repositioned (Figure 4A). Postoperative CT scans (Figure 4B) showed good restoration of the articular surface. To ensure sufficient pain therapy and early functional treatment the patient was kept in inpatient care for five more days. He recovered without any further complications.

### Histology

**Figure 5:**
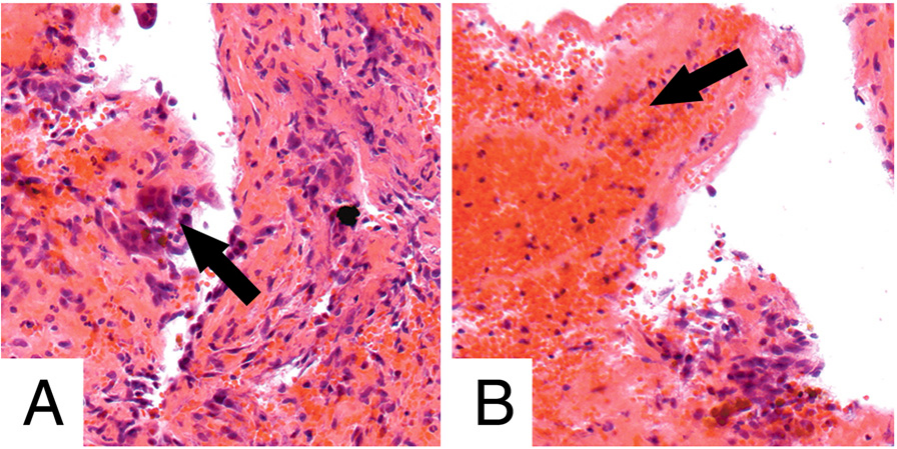
**Histology. A)** The black arrow shows an example of scattered giant cells; **B)** the black arrow shows a fibrinous membrane and some blood cells (elastica van Gieson’s stain).

The histopathological workup of the specimen revealed scattered giant cells and remains of fibrous membranes surrounded by red and white blood cells. There were no signs of any malignant transformation. The lesion was classified as a unicameral bone cyst (UBC). Two representative histological sections are presented in Figure 5. The collected material was decalcified with ethylenediaminetetraacetic acid (EDTA) and was stained with picric acid and acid fuchsin (elastica van Gieson’s stain).

### Follow up

**Figure 6:**
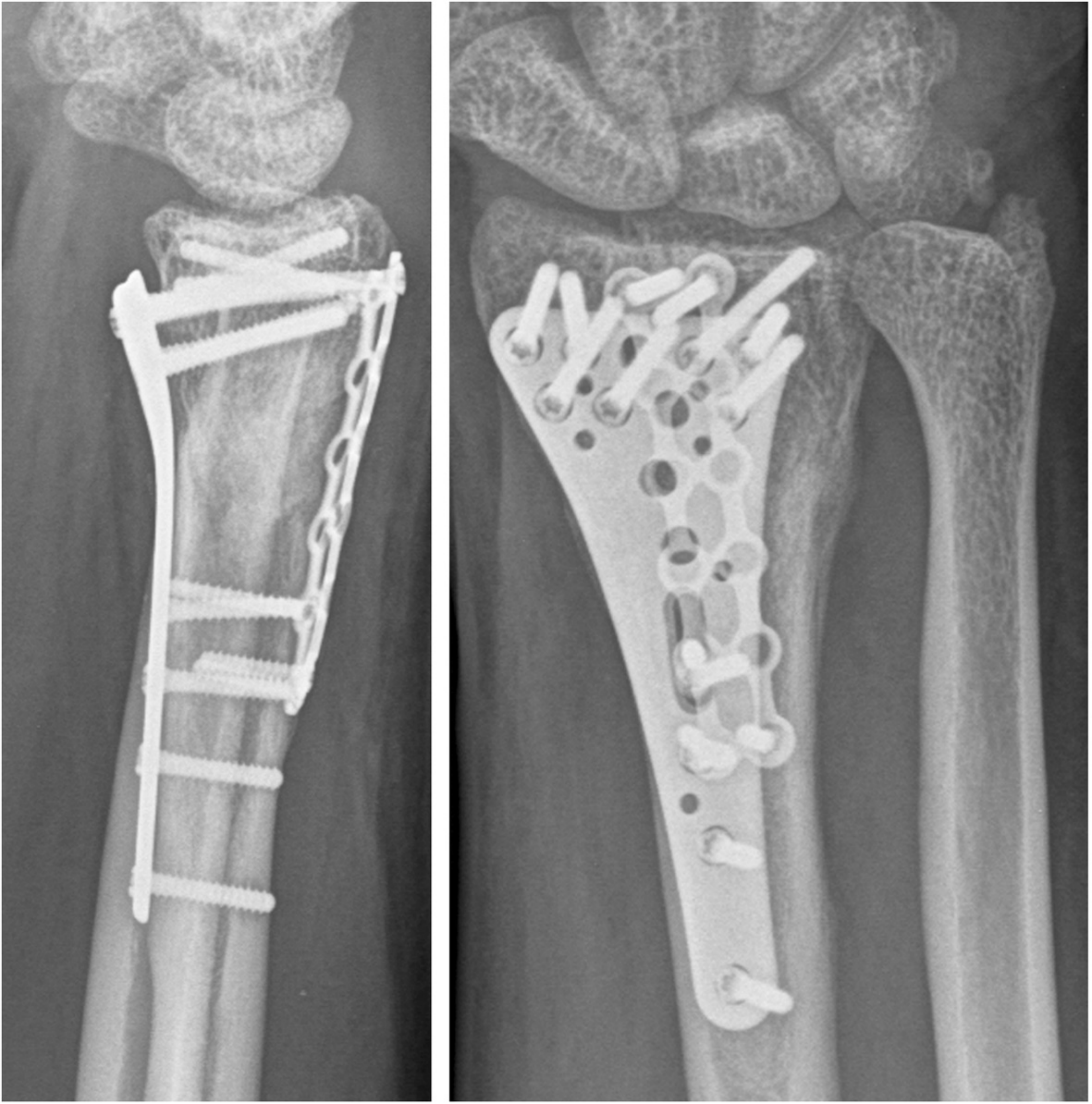
**Radiographs at final follow up 6 months postoperative.** The images show a well reconstructed and anatomically united distal radius.

Regular follow-ups were conducted six weeks, three and six months after the second surgery. Plain radiographs showed a well reconstructed and anatomically united distal radius as well as full bony integration of the autologous bone graft (Figure 6). At six months, the patient was pain-free without restrictions in range of motion or grip strength as compared to the unaffected hand (80° flexion, 80° extension, 30° ulnar deviation, 20° radial deviation, 90° pronation and 85° supination). The Patient-Rated Wrist Evaluation Score(PRWE-Score) [4] was excellent (4 points). The patient returned to work as a cook ten weeks after trauma.

## Discussion

We presented a case report of a fractured, unicameral bone cyst at the distal. The fracture was successfully treated by open reduction, internal fixation and autologous bone grafting. A surgical approach without prior biopsy was chosen based on the patient’s age and the extended radiographic diagnostics revealing a unicameral, cystic lesion with a positive ‘fallen fragment sign’. The ‘fallen fragment sign’ in combination with a unicameral, cystic lesion is almost diagnostic for a UBC [5-9].

The differential diagnosis includes aneurysmatic bone cysts (ABC), giant cell tumors (GCT), non-ossifying fibromas, post-traumatic bone cysts and malignant tumors or metastases. ABC are usually characterized by several blood- or serum-filled cavities of varying diameters. They tend to grow expansively beyond the natural margins of the affected bone thus producing defects within the cortical bone [10]. In plain radiographs, GCT appear as lytic lesions with well-defined but nonsclerotic margins. They are eccentric and also exceed the cortical borders [11]. A definitive diagnosis is obviously only possible upon histopathological examination.

In the literature, the terms juvenile bone cysts or simple bone cysts are synonymously used for UBC. They are unicameral, expansively growing, osteolytic, non-tumorous bone lesions not affecting the cortical bone. UBC are rare, accounting for only 5% of all non-malignant pathological fractures [6,8]. Typical locations are the metaphyseal-diaphyseal region of long bones. The most commonly affected bones are the humerus (23-70%), the femur (23-33%), the calcaneus (11%), the tibia (11%) and the pelvis (10%) [12,13]. Only two papers mention UBC at the distal radius (less than 2,5%) [12,13]. To the authors’ best knowledge, this is the first detailed report on an UBC at the distal radius causing a pathologic DRF in an adult patient.

UBC are usually treated conservatively as they heal spontaneously with skeletal maturity. Although their etiology is unknown, the venous obstruction theory is one of the most accepted models [14,15]. Selected cases, dependent on the location and size of the cyst, may require prophylactic treatment in order to prevent fracture and related complications. For those cases, a variety of treatment approaches has been recommended, including protective bracing, aspiration, local steroid injection, ethanol cauterization, open curettage and bone-grafting or continuous decompression [13,16,17]. Overall, healing rates range between 12% and 92% [12,13,16,17]. There is almost no recommendation for treatment of fractures due to UBC in the literature. Hagmann et al. [18] in 2011 retrospectively analysed 46 cases of UBC, 21 of which resulted in a pathological fracture. UBC were treated either by curettage and bone grafting, corticoid instillation or decompression using cannulated screws with an overall recurrence rate of 39%. They did not specify their treatment regimen for pathologic fractures.

The pathologic fracture presented here required open reduction and internal fixation. The UBC was addressed by curettage and autologous bone grafting harvested by RIA from the ipsilateral femur. Because of the cyst-size and the resulting bone defect we reasoned that filling the defect was essential to ensure stability. Autologous bone graft was chosen because it possesses biomechanical advantages over synthetic bone substitutes and carries no risk of immunogenic reaction or transmission of infectious diseases in contrast to cadaveric allografts or xenografts [19,20].

Intramedullary autologous bone grafts have been shown to yield comparable results regarding stiffness, bone healing and osteoinductive factors when compared to bone grafts from the iliac crest [21]. In a recent prospective study, Sagi et al. [22] showed reduced donor site morbidity for RIA compared to iliac crest grafts. Only limited data is available on how autologous bone grafting harvested by RIA promotes fracture healing [21].

Retrospectively, arthroscopically assisted fracture repair may have prevented the need for revision surgery. To rule out SL-ligament tears, dynamic arthroscopic testing is a validated method [23]. Arthroscopically assisted DRF repair was shown to significantly improve the quality of reduction of the joint surface compared to fluoroscopically guided reduction [24]. Yet to date, no study has proven that arthroscopically assisted fracture repair in DRF results in better long-term clinical outcome. Although there is not enough evidence to support this hypothesis, it seems reasonable to consider wrist arthroscopy whenever difficult intra-articular fragments or co-pathologies such as SL-ligament tears are suspected [25,26].

## Conclusions

A surgical approach without prior biopsy is a reasonable strategy to treat pathologic fractures secondary to UBC, especially if the ‘fallen fragment sign’ is suggesting a benign lesion. Curettage and filling with autologous intramedullary bone graft harvested from the ipsilateral femur by a RIA system yielded an excellent result. Depending upon the nature of the fracture, a combined dorsal and volar approach may be necessary. Arthroscopically assisted fracture repair may be considered in intra-articular fractures or whenever intra-articular co-pathologies such as SL-ligament tears are suspected.

## Consent

Written informed consent was obtained from the patient for publication of this case report and any accompanying images. A copy of the written consent form from our university hospital is available for review by the Editor of this journal.

## Competing interests

The corresponding author confirms that there are no competing interests.

## Authors’ contributions

FM drafted and wrote the manuscript, wrote the revision, did the literature research and made all the follow up examinations of the patient and the documentation of all data. SFB assisted drafting and writing the manuscript, assisted in writing the revision and was responsible for informed consent of the patient. EV was responsible for reviewing the manuscript and substantially contributed to the structure of the manuscript. He finally proof-read and edited the manuscript together with a native English speaker. WM was responsible for the final revision of the manuscript and contributed lots of knowledge and experience concerning cyst-like bone tumors to the case report. SG revised the manuscript and was basically involved in treating the patient and initiating the case report. All authors read and approved the final manuscript.

## Pre-publication history

The pre-publication history for this paper can be accessed here:

http://www.biomedcentral.com/1471-2474/15/202/prepub
